# POCUS Finding of Portal Venous Gas: An Unusual Consequence of an Amyloid Dysmotility Related Bezoar

**DOI:** 10.24908/pocus.v7i2.15681

**Published:** 2022-11-21

**Authors:** Grace B DeMarco, Qiuchen Jiang, Ernest A Fischer

**Affiliations:** 1 Department of Medicine, MedStar Georgetown University Hospital Washington, DC; 2 Department of Pediatrics, MedStar Georgetown University Hospital Washington, DC

**Keywords:** POCUS, Portal gas

## Abstract

A 73-year-old male with a recent finding of pericardial effusion and syncope was evaluated with point of care ultrasound for recurrent effusion. A thickened left ventricle and recurrent pericardial effusion were found. Unexpectedly, on scanning the inferior vena cava (IVC), extensive portal venous gas was identified, a finding previously described as a “meteor shower”. Subsequent imaging by computed tomography (CT) identified gastric edema and peri-gastric vessel gas as the source of the portal gas, attributed to a large bezoar. The bezoar was later classified as a phytobezoar and the patient was found to have both cardiac and gastrointestinal manifestations of light chain amyloidosis. The gastrointestinal amyloidosis predisposed the patient to bezoar formation owing to associated dysmotility, a rare complication of an unusual manifestation of systemic amyloid.

## Case Report

A 73-year-old male with diabetes and hypertension had recently been admitted to our hospital for syncope. Initial work-up revealed a moderate pericardial effusion thought to be partially responsible for the syncope. He underwent pericardiocentesis to remove 300 milliliters of exudative fluid, but testing revealed no evidence of malignancy. There were no signs of recurrence during monitoring and he was discharged with an “idiopathic” effusion.

He returned less than two months later after suffering an episode of near syncope after standing that caused him to crawl on the floor to call for help. On review of systems, he described poor oral intake, worsening early satiety and post-prandial nausea. He noted fruits, particularly peaches, were the only foods he could tolerate that would not lead to significant, uncomfortable fullness. Additionally, he described loose stools following each meal such that he was having two to three stools daily. 

**Figure 1:**
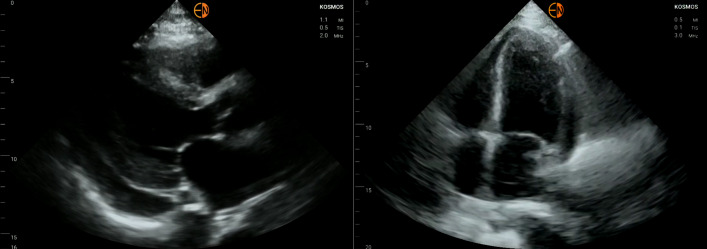
Parasternal long axis (left) and apical four chamber view (right). The left image shows a moderate sized pericardial effusion. In both images the walls were thickened and function was depressed towards the base of the heart. A subsequent echocardiogram described the findings as concerning for amyloid cardiomyopathy.

**Figure 2:**
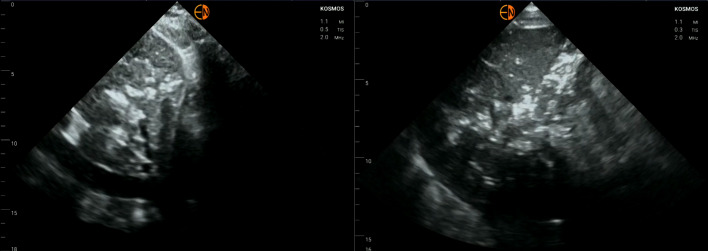
The left image shows the patient’s IVC, which was plethoric, but also notable for scattered hyperechoic areas. The hyperechoic areas were confirmed in subsequent views of the liver (right) and lead to further investigation, as they are indicative of extensive portal venous gas.

Given the history of pericardial effusion we performed a point of care ultrasound (POCUS) of the heart, which showed a recurrent, moderately sized, simple effusion (Figure 1). There was no evidence of tamponade physiology such as any chamber collapse. Despite the low concern, the IVC was assessed as a plethoric IVC, or one that is dilated (>2.1 cm) with <50% respiratory variability, can be expected in >90% of tamponade cases  1. The IVC had a normal size and respiratory variability. Quite striking, however, was the appearance of the surrounding liver, which had diffuse hyperechoic areas confirmed on multiple views (Figure 2). 

**Figure 3:**
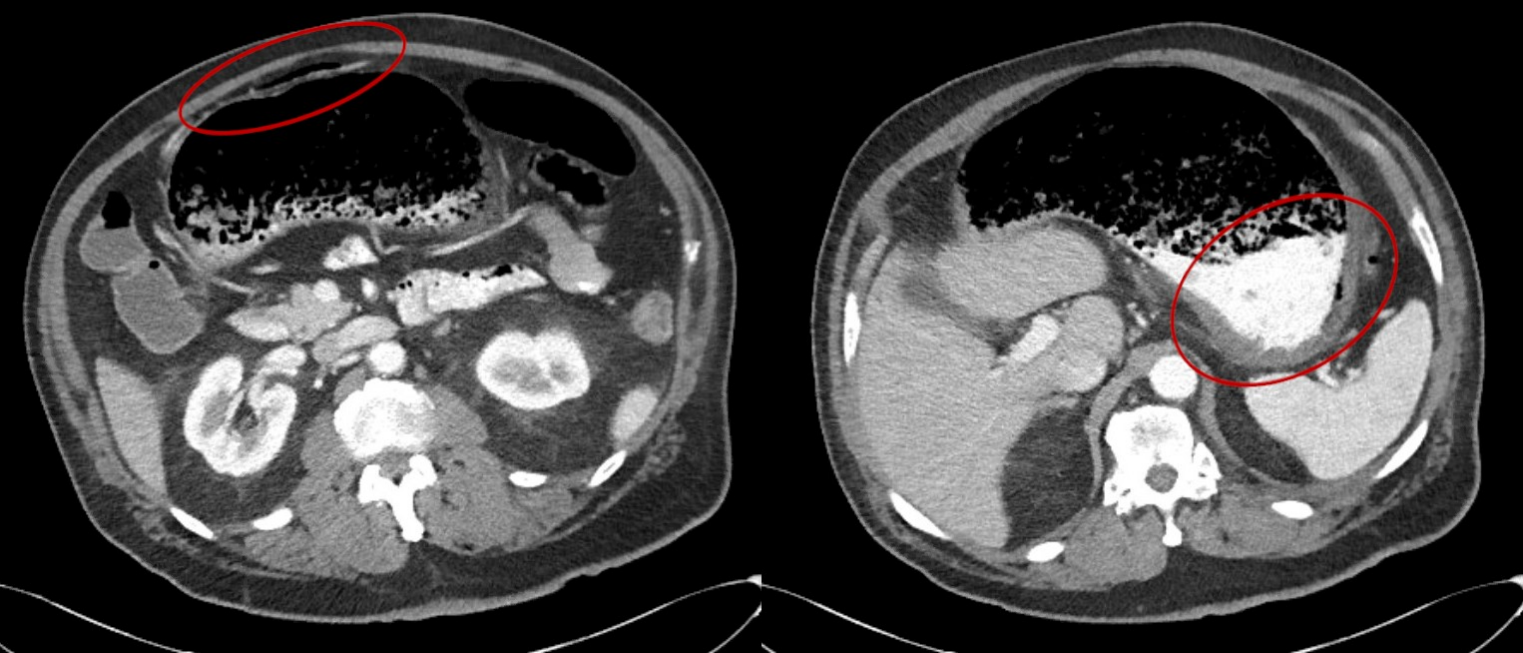
CT Abdomen showing gastric wall edema and gas in the peri-gastric vessels, some of which is highlighted in red.

**Figure 4:**
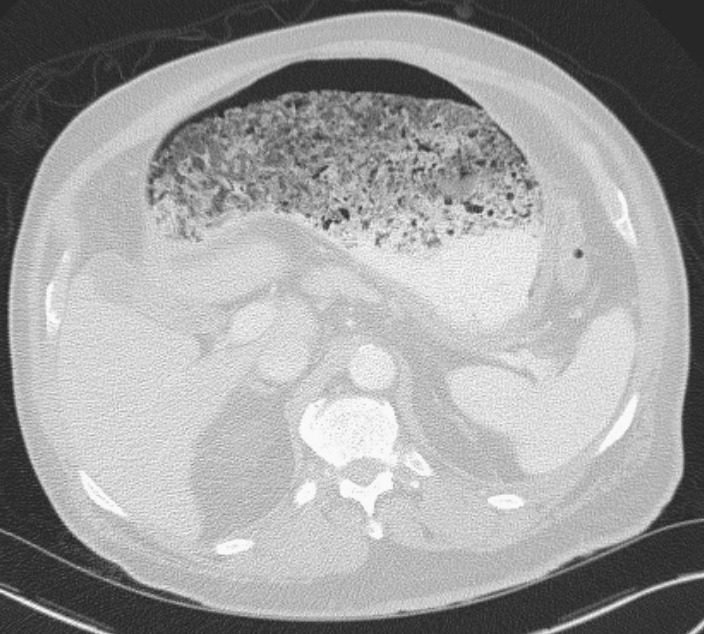
CT Abdomen showing the large amount of gastric contents consistent with a bezoar, later confirmed to be a phytobezoar.

The patient was reassessed and confirmed to have minimal abdominal pain and his abdominal exam was benign. After confirming the finding with radiology, the patient underwent a CT of the abdomen and pelvis. That CT showed gastric wall edema and gas in the peri-gastric vessels as the source of the portal venous gas (Figure 3). A large gastric bezoar was also identified as potentially causal (Figure 4).

A nasogastric tube was placed and cola was infused through it for 48 hours along with administration of scheduled IV metoclopramide as a pro-motility agent. Repeat imaging showed no appreciable change in the bezoar. The patient then underwent endoscopy at which time the bezoar was identified as a phytobezoar. Attempts to remove or fully fragment the phytobezoar were unsuccessful but gastric and duodenal biopsies were taken. These biopsies were both consistent with amyloid, with Congo red staining positive around the lamina propria. Additionally, a cardiac MRI was consistent with cardiac amyloid, Tc-99m PYP scintigraphy made transthyretin (ATTR) amyloidosis unlikely, and electrophoresis showed a monoclonal spike of free lamba protein. 

## Discussion

The scattered hyperechoic areas on the liver in this case have been previously described as a “meteor shower” and are suggestive of portal venous gas 2. Portal venous gas can be non-clinically meaningful, a known result of multiple procedures, intra-abdominal or endoscopic 3. Our patient had not reported any recent procedure known to cause portal gas, thus we were concerned for an acute, non-iatrogenic cause. The patient’s exam made bowel necrosis and ischemia, small bowel obstruction, gastrointestinal ulceration, cholecystitis, cholangitis, enterocolitis, or appendicitis, to name a few, unlikely 3.

The ultimate finding of a bezoar was unexpected. A bezoar is a collection of indigestible material trapped within the gastrointestinal tract, most commonly the stomach. Phytobezoars are composed of plant-based material like cellulose, tannin, and lignin which are found in high concentrations in unripe vegetables and fruits 4. Trichobezoars contain hair, pharmacobezoars result from enteric-coated or long-acting medications, lactobezaors arise from concentrated infant formula, and other bezoars develop from synthetic material like vinyl gloves, cement, and styrofoam 5. 

Though bezoars can form in the absence of underlying gastrointestinal pathology, the vast majority, up to 93%, are related to some predisposing risk factors, such as prior gastric surgery, vagotomy leading to lower gastric acid concentrations, and gastrointestinal dysmotility 6. Gastrointestinal amyloidosis and associated dysmotility may have been the predisposing risk factor in this patient. In patients with systemic amyloidosis, only 1% develop gastric related symptoms and only a fraction of those develop gastroparesis 7. Additionally, gastrointestinal involvement is reported more commonly in AA amyloidosis, in up to 60% 8, compared to only 8% in AL amyloidosis 9, the type from which our patient suffered. 

Treatment of bezoars, especially of phytobezoars, typically begins with conservative management if there are no severe gastrointestinal complications such as obstruction or severe bleeding. Medical management involves chemical dissolution, most commonly with cola 5 and can be coupled with pro-kinetic therapy like metoclopramide to improve gastric motility, which has been shown to improve efficacy of subsequent endoscopic intervention 10. Endoscopy may be necessary when the initial chemical dissolution is unsuccessful to fragment the bezoar into small segments so that they can either be physically pushed past the pylorus or suctioned up via the endoscope. When medical and endoscopic therapy fail, surgical removal can become necessary 5. 

In addition to the gastrointestinal manifestations of amyloidosis, our patient was found to have features of cardiac amyloid on echocardiogram and subsequent cardiac MRI and technetium-99m pyrophosphate (Tc-99m PYP) scintigraphy were obtained. Echocardiographic findings suggestive of cardiac amyloid include ventricular wall thickening, atrial enlargement, thickened valve leaflets, and pericardial effusion; however, these features are not specific for amyloid, though the findings on cardiac MRI and Tc-99m PYP scintigraphy can be 11, 12. Tc-99m PYP scintigraphy uses the bone-seeking nuclear biomarker pyrophosphate to detect calcium uptake in the cardiac myocytes and is used to distinguish ATTR amyloidosis from light chain (AL) amyloidosis 13. Our patient’s Tc-99m PYP scintigraphy had minimal uptake of the radiolabeled tracer, making ATTR amyloid unlikely, and when combined with the cardiac MRI, his cardiac presentation was most consistent with AL amyloidosis, which was subsequently confirmed by electrophoresis.

POCUS did not reveal the source of the immediate portal venous gas, nor were findings of chamber wall thickening and effusion on cardiac ultrasound sufficient to suggest amyloidosis or infiltrative disease, alone 11, 12. We identified a case report^14^ and a case series^15^ with bezoars associated with portal venous gas. In those reports, the patient presented with intussusception or small bowel obstruction, presentations that are known to lead to portal venous gas. In our case, we did not identify an acute abdominal pathology directly responsible for the finding and after one evening of full bowel rest the patient’s portal gas resolved. Despite being a seemingly incidental finding, the portal gas seen on POCUS did lead to a clinical inquiry that resulted in a timely diagnosis for an unusual presentation of phytobezoar and AL amyloid. We believe this case is an archetypal example that demonstrates how in practice and with POCUS, “chance favors the prepared mind” 16.

## Conclusion

This case demonstrates the utility of POCUS in that even an incidental finding (i.e. extensive portal venous gas made at the bedside on presentation) can expedite a clinical workup. This finding led to the diagnosis of a phytobezoar as a result of AL amyloidosis with cardiac and gastrointestinal involvement. This case also highlights a bezoar as a rare manifestation of dysmotility, which is a rare complication of amyloidosis. Our patient, after completion of a cardiac, gastrointestinal, and hematologic work-up, was discharged home hemodynamically stable and tolerating a regular diet, though the bezoar was only partially fragmented. He will require close follow-up with plans for outpatient monitoring of the bezoar with gastroenterology, cardiology for the cardiac amyloid, and hematology for the systemic AL amyloidosis.

## Statement of Consent

The patient provided verbal consent to describe his case presentation in a de-identified manner.

## Disclosures

None.
